# Ontology-Based Modelling and Analysis of Sustainable Polymer Systems: PVC Comparative Polymer and Implementation Perspectives

**DOI:** 10.3390/polym17192612

**Published:** 2025-09-26

**Authors:** Alexander Chidara, Kai Cheng, David Gallear

**Affiliations:** 1 Department of Mechanical and Aerospace Engineering, College of Engineering, Design and Physical Sciences, Brunel University of London, Uxbridge UB8 3PH, UK; 2 Brunel Business School, Brunel University of London, Uxbridge UB8 3PH, UK; david.gallear@brunel.ac.uk

**Keywords:** ontology, polymer sustainability, circular economy, polyvinyl chloride (PVC), semantic reasoning, sustainable materials, recycling technologies

## Abstract

This study develops an ontology-based decision support framework to enhance sustainable polymer recycling within the circular economy. The framework, constructed in Protégé (OWL 2), systematically captures polymer categories with emphasis on polyethylene terephthalate (PET), polylactic acid (PLA), and rigid polyvinyl chloride (PVC) as well as recycling processes, waste classifications, and sustainability indicators such as carbon footprint. Semantic reasoning was implemented using the Semantic Web Rule Language (SWRL) and SPARQL Protocol and RDF Query Language (SPARQL) to infer optimal material flows and sustainable pathways. Validation through a UK industrial case study confirmed both the framework’s applicability and highlighted barriers to large-scale recycling, including performance gaps between virgin and recycled polymers. The comparative analysis showed carbon footprints of 2.8 kg CO_2_/kg for virgin PET, 1.5 kg CO_2_/kg for PLA, and 2.1 kg CO_2_/kg for PVC, underscoring material-specific sustainability challenges. Validation through a UK industrial case study further highlighted additive complexity in PVC as a major barrier to large scale recycling. Bibliometric and thematic analyses conducted in this study revealed persistent gaps in sustainability metrics, lifecycle assessment, and semantic support for circular polymer systems. By integrating these insights, the proposed framework provides a scalable, data-driven tool for evaluating and optimising polymer lifecycles, supporting industry transitions toward resilient, circular, and net-zero material systems.

## 1. Introduction

Polymers are of significant industrial and commercial interest, but their environmental impact has raised growing concerns [1]. While valued for durability, affordability, and versatility, issues such as chlorine content, additive complexity, and limited recyclability have cast doubts on their sustainability [2]. The polymer industry underpins modern manufacturing, with applications across packaging, construction, automotive, and electronics. However, both fossil-based polymers—such as polyvinyl chloride (PVC), polyethylene terephthalate (PET), polypropylene (PP), polystyrene (PS), and carbon fibre reinforced polymer (CFRP)—and bio-based alternatives like polylactic acid (PLA) and polyethylene (PE) face critical ecological challenges [3]. The linear “take–make–dispose” model contributes to resource depletion, greenhouse gas emissions, and plastic pollution, making the adoption of circular-economy (CE) strategies imperative [4].

Conventional recycling methods are limited by non-standardised practices, poor interoperability, and degradation of material quality [5]. PVC is widely used due to durability and cost-effectiveness, but recycling is hindered by additive contamination and risks from dehydrochlorination [6]. PET demonstrates higher recyclability but suffers from performance loss and microplastic release during mechanical recycling. CFRP, applied extensively in aerospace, maritime, automotive, and construction sectors for its strength and lightweight properties, generates substantial waste, with recyclability only recently advancing through thermal recycling [7]. These challenges underscore the need for comprehensive evaluation frameworks that integrate chemical, environmental, and economic factors [8].

**Figure 1:**
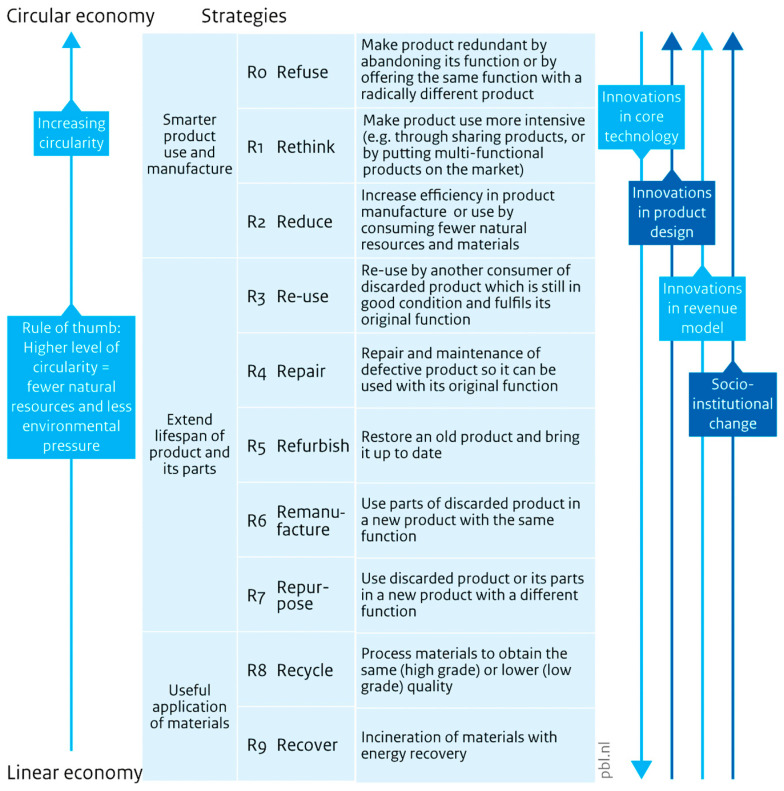
The 9R Framework of CE [9].

The transition from linear to circular polymer systems is central to advancing material sustainability. The 9R framework (Refuse, Rethink, Reduce, Reuse, Repair, Refurbish, Remanufacture, Repurpose, Recycle, Recover) provides a hierarchical pathway from waste management to high-level resource efficiency shown in Figure 1 [9].

As illustrated, the framework exhibits multiple transition steps from the linear economy to CE, although polymers are now essential for modern industries and they repeatedly encounter ecological issues related to their fossil origin, additive content, and end-of-life limitations [10].

Recent studies illustrate its application in polymer systems: PET recycling is advancing through closed-loop bottle-to-bottle processes [11], while PVC remains largely constrained to recycling and recovery due to additive complexity [2]. Integration of chemical and biological catalysis offers new opportunities for upcycling and reduced energy demand [6]. Multi-R innovation strategies are increasingly emphasised in sustainable polymer development [12].

Ontology-based modelling provides a structured solution by representing domain knowledge in machine-readable formats, enabling semantic reasoning and integration of diverse datasets. This work introduces a novel ontology for sustainable polymer systems, focusing on three representative materials—PET, PLA, and PVC—selected for their distinct sustainability profiles and industrial relevance [13].

### 1.1. Industrial Significance of Sustainable Polymers and Circular Economy

Global plastic production exceeds 380 million tonnes annually [14], yet only 9% of waste is recycled, the majority is incinerated or discarded into ecosystems [15,16].

The global polymer industry, valued at USD1.2 trillion in 2024, faces increasing pressure to transition from linear to circular models [14,17,18]. For example, PVC’s chlorine chemistry reduces fossil feedstocks demand by 43%, but also complicates recycling through dehydrochlorination [13]. With 40 million tonnes of PVC produced annually, enhancing its recyclability could lower construction related carbon emissions by 15–20% [13,19]. Notably, 60% of mechanical recycling failures are attributed to additive contamination [20]. CE strategies therefore aim to decouple economic growth from raw material consumption through reuse, high-quality recycling, and substitution with more sustainable feedstocks [21].

**Figure 2:**
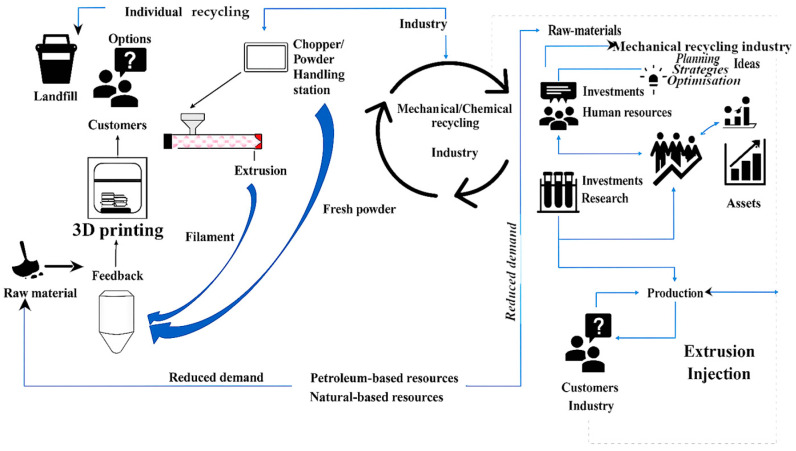
Illustration of advanced sustainability through the interrelationships among polymer production, recycling strategies, and the circular economy [22].

Figure 2 illustrates CE principles applied to polymers, highlighting the relationship between production, recycling strategies, and sustainability [22]. It emphasises the need to reduce reliance on fossil-based feedstocks while adopting efficient, high-quality alternatives.

### 1.2. Research Gaps and State-of-the-Art Overview

Despite advances in mechanical, chemical, and enzymatic recycling, persistent barriers remain. Practical issues include additive incompatibilities, performance degradation, and weak integration of recyclability into lifecycle assessments. Research challenges include:

- Fragmented sustainability metrics: In total, 78% of lifecycle assessments (LCAs) use incompatible indicators, hindering cross-comparisons [3,23].
- Ontological gaps: No semantic framework comprehensively links polymer chemistry, recycling routers, and sustainability strategies.
- Weak industrial validation: Only 12% of CE models are tested against real-world production data [24].
- Inadequate modelling of CE dynamics: Existing ontologies (e.g., MPSeP frameworks) lack polymer-specific reasoning capacity, for material flows and recycling pathways.

Moreover, while ontology-based reasoning and AI-driven modelling have advanced in energy systems, applications in polymer circularity remain sparse [25]. Bibliometric studies show rising attention to AI-enabled sorting, chemical recycling, and bio-based plasticisers, but they also highlight the absence of integrated semantic frameworks to guide decision-making [26,27,28].

Accordingly, this paper develops an ontology-based decision-support framework that systematically integrates polymer categories, recycling processes, sustainability indicators, and environmental impact data into a unified knowledge system. Unlike existing sustainability ontologies, which remain fragmented across domains such as energy, agriculture, or construction, this work explicitly addresses polymer systems and their life cycles, including both fossil-based (PVC, PET) and bio-based (PLA) materials. The practical significance of the framework lies in its ability to combine semantic reasoning (through SWRL rules and SPARQL queries) with industry-relevant simulations (using ANSYS Granta EduPack 2023 R2 Version: 23.2.1), thereby enabling reproducible and data-driven evaluation of polymer sustainability. This integration provides decision-makers and industry stakeholders with a validated tool to compare trade-offs, identify recycling barriers, and guide circular-economy transitions in practice.

### 1.3. Research Questions and Objectives

In doing so, the study explored the following questions:

1. How can ontology-based modelling be effectively employed to represent and facilitate reasoning about complex circular economic dynamics in polymer systems?
2. What are the sustainability trade-offs and circularity potentials of commonly used polymers such as PVC, PLA, and PET when evaluated through simulation tools and semantic models?
3. What barriers and opportunities exist in industrial PVC recycling workflows, and how can these be addressed using semantic reasoning and digital integration?
4. To what extent do current academic and industrial discourses align with the sustainability, degradation, and recyclability of bio-based and fossil-based polymers?
5. How can semantic and simulation-based frameworks support the transition toward net-zero goals in polymer manufacturing and design?

To address these questions, our goals are:

1. To design and implement a semantic ontology model in Protégé (OWL 2) that captures essential circular-economy (CE) parameters for polymers, including material types, recycling methods, environmental impacts, and sustainability metrics.
2. To integrate semantic reasoning using SWRL and SPARQL for inferring optimal material flows and circular pathways, addressing recyclability, embodied energy, and carbon footprint.
3. To perform a comparative simulation of polymer sustainability performance (including PVC, PLA, and PET) using ANSYS Granta EduPack for material selection and trade-off analysis.
4. To validate the ontology framework through an industrial case study in the UK PVC manufacturing sector (General Manufacturing of Windows and Doors), focusing on pre- and post-consumer waste streams, recycling constraints, and CE barriers.
5. To conduct a bibliometric and thematic analysis using VOS viewer and Biblioshiny to identify knowledge gaps, trends, and research priorities in sustainable polymer circularity.
6. To explore implementation perspectives by assessing the integration of the proposed framework into industrial digital tools (ERP, IoT, Digital Twins) and discussing practical implications for manufacturing, lifecycle planning, and policy design.

The rest of the paper is structured as follows: Section 2 presents methods; Section 3 details the ontology model; Section 4 describes the industrial case study; Section 5 presents results; Section 6 discusses implementation; Section 7 concludes with contribution and future research directions.

## 2. Materials and Methods

The methodology was designed to be reproducible and transferable, enabling other researchers to replicate the processes of ontology construction, semantic reasoning, simulation modelling, and industrial case study validation.

### 2.1. Purpose

This study addresses a key research gap by developing a novel ontology for sustainable polymer systems in Protégé (OWL2) (version 5.6.3, Stanford Center for Biomedical Informatics Research (BMIR), Stanford, CA, USA), structured around circular-economy (CE) parameters. The ontology models major polymer classes PVC, PET, and PLA using semantic reasoning to represent lifecycle loops and support sustainability-oriented decision-making.

### 2.2. Scope

The framework integrates SWRL and SPARQL reasoning with sustainability indicators, recycling pathways, and waste classifications. PET, PLA, and rigid PVC were chosen as representative case materials because of their distinct sustainability profiles, industrial relevance, and environmental impact. The design also responds to bibliometric gaps in lifecycle assessment and semantic modelling.

### 2.3. Ontology Function and Reasoning Approach

SWRL rules and SPARQL queries are applied to infer material flows and identify sustainable pathways. Comparative simulations and trade-off analyses are performed with ANSYS Granta EduPack, providing a unified platform for evaluating polymer sustainability and enabling industrial validation.

### 2.4. Intent and Validation

A UK-based industrial case study serves to validate the framework, highlighting recycling barriers and performance differences between virgin and recycled polymers. The findings demonstrate the practical relevance of the approach and its potential to guide industry in advancing toward resilient and sustainable material lifecycles.

## 3. Ontology-Based Modelling and Semantic Reasoning Framework

Addressing circularity and sustainability in polymers requires tools that can integrate diverse data types and support precise, reproducible decision-making. Ontology-based modelling formalises polymer lifecycle knowledge in a machine-readable format and is therefore well suited to this task. We developed a semantic reasoning framework centred on major polymers and validated it using data from a UK PVC manufacturer. In the process, some qualitative and commercially sensitive information was withheld for confidentiality.

### 3.1. Ontology Design in Protégé (OWL 2)

#### 3.1.1. Design Rationale

Ontology frameworks advance quantitative polymer analysis especially for large heterogeneous datasets by structuring domain knowledge to improve data collection, modelling, and decision support. The wide range of polymer applications complicates measurement of production volumes and environmental impacts, thereby necessitating the structured approach presented here.

#### 3.1.2. Class Hierarchy

The ontology, implemented in Protégé (OWL 2), classifies polymers and encodes associated object and data properties and subclasses. Distinguishing thermoplastics from biodegradable polymers is essential for comparative analysis because these groups differ in functional application, environmental behaviour, and sustainability profile [29,30].

The primary material-centric hierarchy is:

- Thermoplastics: polyvinyl chloride (PVC), polyethylene terephthalate (PET), polypropylene (PP), high-density polyethylene (HDPE), low-density polyethylene (LDPE).
- Biodegradable polymers: polylactic acid (PLA), polyhydroxyalkanoates (PHA), polybutylene succinate (PBS).

This structure enables systematic organisation of polymer attributes processing methods, molecular architecture, physical behaviour, and environmental impacts thereby supporting lifecycle assessment and sustainability evaluation.

#### 3.1.3. Properties and Sustainability Metrics

Key physical and chemical properties are incorporated into ontology to define polymer performance across applications. These properties include:

Mechanical Properties
Thermal Properties
Chemical Resistance
Biocompatibility [29,30].

It also incorporates environmental footprint data and a dedicated sustainability domain that includes:

Carbon Footprint
Energy Consumption
Degradation Time
Renewable Resource Utilisation [31,32].

In summary, the ontology provides a robust, extensible, and semantically rich infrastructure for formalising polymer sustainability knowledge, enabling automated reasoning and data-driven decision support for circular-economy applications.

### 3.2. SWRL Rules and SPARQL Queries

The Protégé (OWL 2) ontology implements semantic reasoning using the Semantic Web Rule Language (SWRL) and supports structured retrieval via the SPARQL Protocol and RDF Query Language (SPARQL). SWRL rules encode domain knowledge and enable the ontology to infer implicit relationships such as recyclability, compatibility constraints, and sustainability trade-offs while SPARQL queries extract complex, structured information on material relationships, lifecycle stages, and sustainability indicators to support decision-making.

Material data were populated from ANSYS Granta EduPack Level 3 [33], which provides datasets for over 2000 polymer materials (mechanical, thermal, biodegradability and other sustainability metrics). Material selection combined datasheet review with sustainability criteria to enable trade-off analysis; simulation outputs were exported and organised into a comparative table for simultaneous evaluation of twenty candidate materials [33].

SWRL and SPARQL were applied iteratively: SWRL rules inferred new relationships and candidate insights, and SPARQL queries retrieved and filtered results for scenario analyses and reporting. Together they provide an active reasoning and data-retrieval layer within the decision-support platform, enabling reproducible material selection and sustainability assessment.

#### 3.2.1. SWRL Rule Development

Within the Protégé environment, SWRL rules enable logical deduction by inferring new knowledge from existing ontology definitions. For instance, a rule can classify a polymer as sustainable if it combines high recyclability with a low environmental footprint:

Polymer (? p) ∧ is Recyclable (? p, true) ∧ has Greenness (? p, low) → is Sustainable (? p, true).

This automated reasoning allows the ontology to dynamically update classifications as new data are incorporated, thereby maintaining the accuracy and relevance of sustainability assessments [34].

#### 3.2.2. SPARQL Query Framework

SPARQL queries systematically retrieve insights from the ontology, particularly regarding sustainability metrics. For example, a query can be written to identify all polymers meeting defined sustainability criteria:

PREFIX ex: SELECT? polymers WHERE {? polymers ex: is Sustainable true.}

Such queries enable efficient identification of candidate materials for experimental testing and the development of sustainable alternatives [35].

### 3.3. Simulation-Based Polymer Trade-Off Analysis

To complement the ontology, a sustainability-focused simulation study was conducted using ANSYS Granta EduPack (Material Universe and Eco Audit tools). Six polymers were compared: polyvinyl chloride (PVC), polyethylene terephthalate (PET), polypropylene (PP), polylactic acid (PLA), polyhydroxyalkanoates (PHA), and polystyrene (PS).

Each material was evaluated against circular-economy sustainability criteria, including mechanical and thermal properties, recyclability, biodegradability, cost, carbon footprint, energy use, and end-of-life options. Quantitative data from the simulations provided a robust basis for material trade-off analysis and informed the ontology modelling in Protégé.

**Figure 3:**
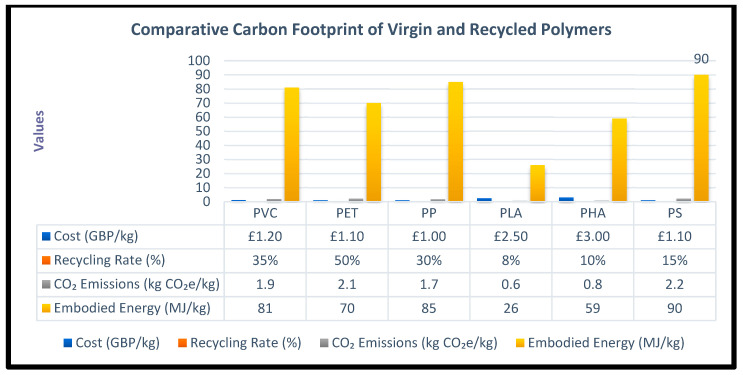
Comparative sustainability metrics for selected polymers [36].

Figure 3 and Table 1 and Table 2 present comparative results.

#### 3.3.1. Material Selection Approach

Material selection used the ANSYS Granta EduPack Level-3 Polymer Sustainability database (>2000 materials). The database’s chart-selection tools screened candidates and six polymers were chosen for trade-off analysis: polyvinyl chloride (PVC), polyethylene terephthalate (PET), polypropylene (PP), polylactic acid (PLA), polyhydroxyalkanoates (PHA), and polystyrene (PS). These candidates were evaluated in the Eco Audit module to quantify recyclability, environmental impacts, cost, and early-stage application suitability within a circular-economy context.

The Eco Audit produced quantitative metrics such as embodied energy and CO_2_ emissions per kilogram covering production, manufacturing, transport, use, and end-of-life stages. Simulation outputs were exported and consolidated into a single Excel workbook to support structured ontology population and subsequent comparative analysis in Protégé.

#### 3.3.2. Literature-Supported Evaluation of Materials

Mechanical, thermal, and electrical properties strongly influence material selection. PVC and PET exhibit high tensile strength and modulus, making them attractive for structural and packaging applications, respectively; PVC’s thermal stability and insulating properties are linked to its chlorine chemistry [37,38], while PET’s melting point and thermal properties suit packaging and textile uses [39]. Biodegradable polymers (PLA, PHA) generally offer environmental advantages but have lower thermal resistance; PP provides a balanced profile with a low glass transition temperature, and PS delivers moderate mechanical strength but limited thermal resistance [40].

Environmental and economic trade-offs differ across the set. PS and PVC typically show higher fossil-derived CO_2_ emissions, whereas PLA and PHA tend to have lower carbon footprints due to their biomass origins [41]. PET benefits from an established recycling infrastructure and relatively energy-efficient mechanical recycling; whereas PVC recycling is complicated by additive complexity. Economically, PVC, PET, and PP are cost-competitive, while PLA and PHA remain relatively expensive despite ecological benefits such as lower embodied energy and industrial-compostable end-of-life properties [42]. As far as recycling-related challenges are material specific: PET recycling is well developed, PVC recycling is complicated by additive complexity, and PS recycling is vulnerable to contamination [37,39].

Combining literature evidence with ANSYS Granta EduPack simulations shows that PET and PVC currently offer the best balance of mechanical performance, cost, and data completeness for ontology modelling. Bio-based polymers (PLA, PHA) show strong long-term potential but require supportive infrastructure and cost reductions. For these reasons, PVC was selected as the central industrial case material, with PET and PLA serving as reference materials for comparative benchmarking within the ontology.

With the ontology structure and semantic reasoning in place, the system was used to quantify circularity and environmental trade-offs for the selected polymers. Industry-specific simulation outputs are presented in Section 5.3 to compare PVC, PLA, and PET and to translate abstract metrics into actionable sustainability insights. The subsequent industrial case study of a UK PVC manufacturing facility (Section 4) validates the modelling results and highlights real-world barriers and opportunities for circular polymer integration.

## 4. Industrial Assessment of PVC Waste Streams and Circular-Economy Readiness in a UK PVC Manufacturing Company: Barriers and Opportunities for Sustainable Polymer Integration

### 4.1. Introduction and Context

This section reports a case study conducted at a UK manufacturer of PVC windows and doors. The company remains anonymous under an agreed confidentiality arrangement; all data were collected onsite with management permission. The primary objective was to provide an empirical basis for ontology-based modelling of sustainable polymer lifecycles, with a focus on PVC relative to alternative polymers. The facility operates semi-automated production across twenty lines and generates substantial volumes of pre-consumer off-cuts and post-production rejects. Observations and measurements were anonymized and aggregated to preserve confidentiality while remaining representative of the broader UK PVC-manufacturing sector [43]. The case study functions as a proof-of-concept for applying ontology-based models in circular-economy settings. We adopted a mixed-methods design that combined quantitative process measurements with qualitative insights from cross-functional stakeholders to enable methodological triangulation. Data collection targeted the following segmented production stages:

1. Raw Material Inbound Logistics & Preparation.
2. PVC Profile Cutting and Machining
3. Frame Welding and Assembly.
4. Hardware Integration.
5. In-Process Quality Control.
6. Glass Unit Fabrication.
7. Glazing & Final Assembly.
8. Final Quality Assurance (QA) and Functional Testing.
9. Packaging & Dispatch.

### 4.2. Waste Management Practices and Circular-Economy Readiness

To evaluate operational practices and organisational readiness, a structured, anonymized questionnaire was completed by six operational staff across production stages. In addition, semi-structured interviews were conducted with five senior staff (three technical managers, one compliance manager, and the plant manager), covering managerial perspectives and decision-making. The instruments assessed workforce awareness, current waste-management practices, perceptions of organisational culture change, and practical barriers to implementing circular-economy measures.

The data collection aimed to (i) characterise how waste is generated, segregated, and recycled across production stages, (ii) identify technical and organisational constraints to recycling and reuse, and (iii) evaluate the plant’s readiness to adopt CE principles and integrate data-driven systems (e.g., ERP, IoT) to sustain product value and improve lifecycle outcomes. Findings from this assessment informed the ontology population and the validation of semantic reasoning against real-world workflows (see Section 3 and Section 5) [44,45].

### 4.3. Quantitative Findings

**Figure 4:**
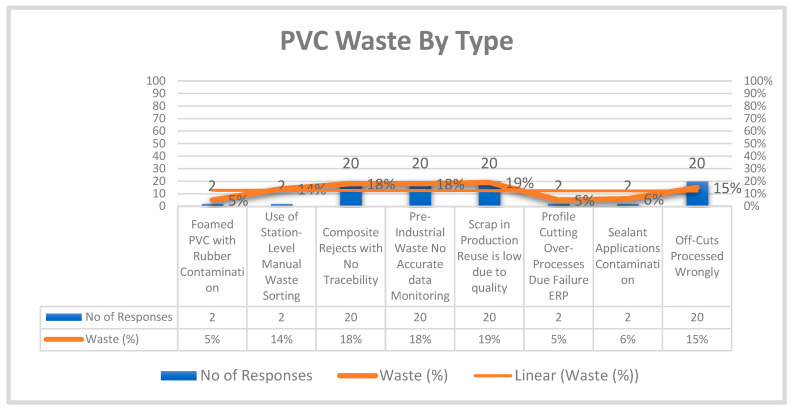
PVC waste by type.

Analysis of the collected data confirms previously reported inefficiencies in polymer manufacturing and validates established circular-economy (CE) models using real production evidence. Both industrial observations and survey data were used to categories’ specific PVC waste streams. Figure 4 illustrates the process-specific distribution of waste, showing substantial material loss during the cutting stage, which directly reduces assembly efficiency [46].

Among the identified categories, composite materials, pre-consumer waste, installation scrap, and off-cuts were the most frequently reported, each cited 20 times. These groups also recorded the highest waste percentages (15–19%), reflecting persistent material loss during cutting, shaping, and fitting. In particular, off-cuts were highlighted as an unavoidable by-product of dimension-specific manufacturing. The alignment between high response frequency and waste percentage indicates recurring inefficiencies across fabrication and installation [46].

By contrast, foamed products, sealants, profiles, and recycled-content applications showed significantly lower waste rates (5–6%), with only two responses each. These materials are either processed more efficiently or used less frequently in the surveyed applications [46].

Composite assemblies, such as PVC combined with rubber seals or other mixed materials, were identified as a major barrier to recycling. Their heterogeneous composition reduces waste stream purity and limits suitability for standard reprocessing, reinforcing earlier literature that highlights composite plastics as a significant challenge for mechanical recycling [46].

Targeted interventions are needed to address these inefficiencies. Design-for-disassembly principles could improve material separation and recycling, while digital cutting optimisation offers potential to reduce off-cuts and improve material yield. Together, these measures could significantly reduce waste generation and advance sustainable material flows [46].

**Figure 5:**
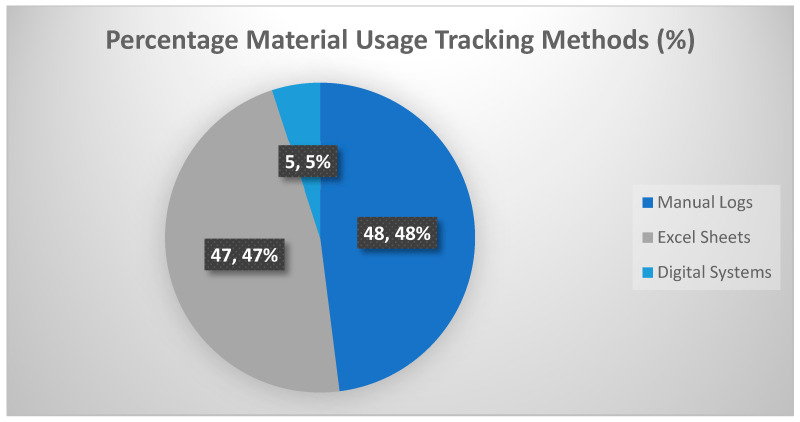
Percentage of how material use and tracking is recorded.

The digital maturity assessment (Figure 5) shows limited use of real-time monitoring and tracking systems, with continued reliance on analogue data collection. This fragmented and error-prone approach restricts accurate diagnosis of waste sources and reflects a low level of Industry 4.0 readiness [47].

**Figure 6:**
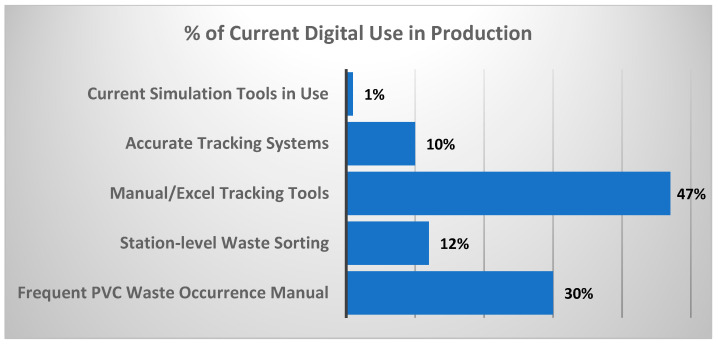
Percentage of current use of digital in the processes.

Complementary findings (Figure 6) reveal minimal adoption of advanced digital tools, with lifecycle simulation software used in less than 1% of cases, leaving a gap in eco-design and lifecycle assessment integration [48]. Weaknesses in digital infrastructure continue to constrain process optimisation and CE adoption in polymer manufacturing.

**Figure 7:**
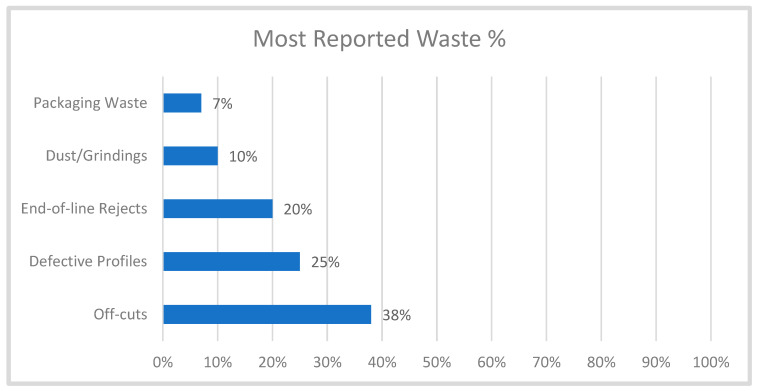
Most reported waste across the processes.

Waste composition profiling (Figure 7) further indicates that losses are concentrated in upstream processes: off-cuts (38%), defective profiles (25%), end-of-line rejects (20%), grindings/dust (10%), and packaging losses (7%). These values are consistent with wider industry reports, showing that early-stage operations—particularly cutting and shaping—are critical points of inefficiency [49]. Factors include over-specification, process tolerances, and reliance on outdated machinery [49].

**Figure 8:**
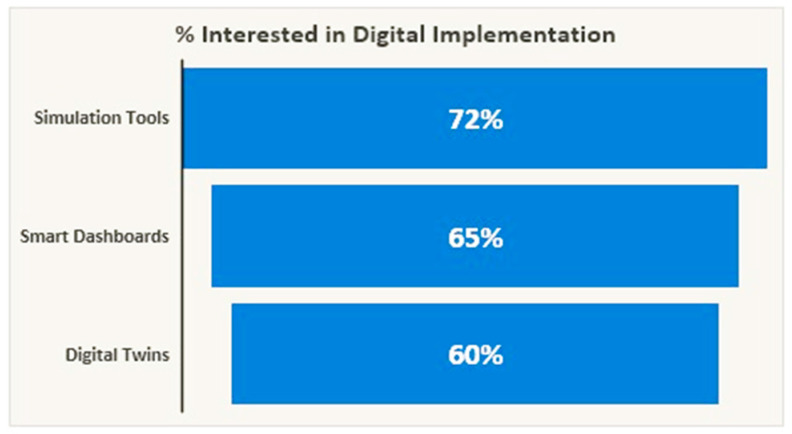
Interest in digital adoption for CE.

Despite these limitations, workforce attitudes toward digitalisation are largely positive. A total of 72% of employees expressed willingness to adopt simulation and dashboard technologies, while 60% supported the implementation of digital twin systems (Figure 8). However, organisational barriers remain, including cost constraints, limited training, IT–operations misalignment, and resistance linked to legacy infrastructure. These systemic challenges echo broader obstacles to digital transformation reported in the literature [50].

### 4.4. Qualitative Insights

Interviews and on-site observations identified inefficient cutting operations, high defect rates, and the absence of comprehensive digital design tools as primary sources of waste. Interviewees confirmed that mixed-material assemblies (for example, PVC combined with rubber seals or strong adhesives) remain a major obstacle to recyclability because they reduce waste-stream purity and complicate mechanical reprocessing. Reliance on manual logs further undermines data traceability and prevents real-time feedback needed for process optimisation; the plant therefore plans to introduce intelligent dashboards and digital-twin capabilities to improve waste visibility and enable predictive management [48].

Key barriers to circular-economy (CE) adoption include limited internal reuse streams, weak digital literacy, inadequate material-separation procedures, and low employee engagement. These organisational constraints map closely to systemic issues reported in the literature: resistance to change, siloed data infrastructures, and coordination inefficiencies, which impede effective CE implementation in polymer production [49].

Interviewees also identified practical opportunities: simulation-based material selection, substitution with more sustainable materials (e.g., PET-G hybrids and bio-based polymers), and deployment of ontology-based semantic models to integrate heterogeneous data and support CE decision-making [48,50]. Semantic modelling can mitigate data fragmentation by establishing logical inference mechanisms that contextualise sustainability indicators across all lifecycle phases, thereby strengthening early-stage eco-design and downstream material-flow decisions.

Taken together, the qualitative evidence highlights both operational and strategic shortcomings that hinder transition to circular polymer systems in UK PVC manufacturing. Despite these challenges, available recycling schemes exist, highlighting the need for coordinated technological, process, and training interventions.

The empirical insights reported here directly informed ontology population and the validation of semantic reasoning (see Section 3 and Section 5). To situate these findings within broader research trends and identify remaining knowledge gaps, a bibliometric and thematic analysis was conducted and its results are summarised in Section 5.5.

## 5. Results and Discussion

### 5.1. Ontology Modelling in Protégé

**Figure 9:**
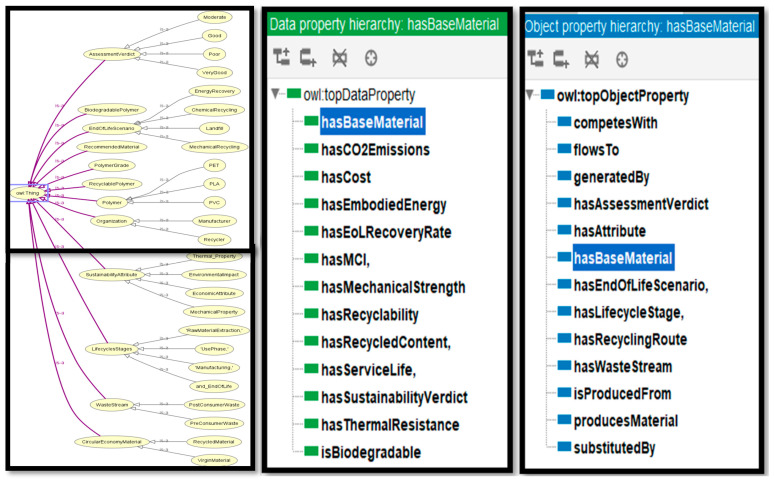
Ontology class domain and data properties for quantitative analysis [51].

The ontology was developed in Protégé (OWL 2) to assess three representative polymers PVC, PET, and PLA selected for their industrial maturity, recyclability, and availability of comprehensive datasets. Using property and class information extracted from ANSYS Granta EduPack, we defined hierarchical classes, subclasses, and associated object/data properties to capture material attributes, processing routes, and lifecycle stages. SWRL rules and SPARQL queries were implemented to enable semantic inference and structured retrieval, and comparative analyses were used to rank candidate polymers according to integrated sustainability metrics. The framework evaluates circular-economy performance using decision metrics such as recycling rate, embodied energy, and net-zero emissions potential, all derived from the merged data sources. Figure 9 illustrates the principal applications and data flows within the ontology-based modelling framework.

### 5.2. Inferred CE Pathways and Recyclability Insights

#### 5.2.1. SWRL/SPARQL Queries Reasoning for Circular-Economy Sustainability Pathways Results

This section describes how the ontology was used to infer circular-economy (CE) pathways and assess recyclability for PVC, PET, and PLA. Key attributes CO_2_ emissions, embodied energy, recyclability, and cost served as the primary decision metrics for comparative evaluation (Table 3).

After defining the class hierarchy, data properties, and inter-class relationships in Protégé (OWL 2), automated reasoning was executed to classify polymers according to the integrated sustainability metrics. SWRL rules (Table 4) encoded domain logic and enabled the ontology to infer new classifications (for example, candidate status for circular reuse or suitability for specific recycling routes).

##### SPARQL Queries & Results

SPARQL queries were then used to extract inferred results and populate the decision matrix for comparative analysis. The dynamically generated classification results are summarised in the table below, illustrating each polymer’s inferred sustainability profile. The matrix can be summarised in Table 5 below.

The SPARQL query outputs from the Protégé ontology generated data-property assertions for PET, PLA, and PVC, respectively. Each polymer instance was linked to quantitative values obtained from ANSYS Granta EduPack and to literature-derived indicators, along with qualitative performance metrics. The results are summarised in Table 5 above [51].

Table 6 provides a consolidated comparative analysis of these SPARQL assertions for PET, PLA, and PVC. It summarises structured semantic characteristics lifecycle stage, mechanical and thermal properties, environmental indicators, and economic metrics used for automated reasoning within the ontology [51].

Consolidating instances into a single comparative table clarifies how semantic properties vary among representative polymers and enables rule-based classification (SWRL) and query-based retrieval (SPARQL).

The combined SWRL/SPARQL and simulation analyses reveal distinct sustainability profiles: PET scores highly on recyclability with moderate energy demand; PLA shows a low carbon footprint and biodegradability advantages; and PVC, despite broad industrial use and production scale, displays higher energy intensity and only moderate recyclability. These results underline the importance of improved waste segregation and advanced recycling routes for PVC to enhance its circular-economy potential.

#### 5.2.2. Summary of the SPARQL-Query Analysis (Protégé Ontology)

Ontology-based reasoning (SWRL + SPARQL) ranks PLA as the most sustainable of the three polymers examined. The ontology outputs indicate that PLA has the lowest CO_2_ emissions (1.2 kg CO_2_e/kg), the lowest embodied energy (55 MJ/kg), and is biodegradable; it is assigned a “Very Good” sustainability rating and classified as a Low-Carbon Polymer.

PET, although non-biodegradable, scores highly for recyclability, shows moderate embodied energy (70 MJ/kg), and has a low material cost (£1.0/kg). The ontology classifies PET as a Low-Carbon Polymer with a “Good” sustainability verdict making it attractive for closed-loop recycling though not meeting the “Recommended Material” threshold in this analysis.

PVC records the highest CO_2_ emissions (2.0 kg CO_2_e/kg) and embodied energy (80 MJ/kg), with only moderate recyclability and no biodegradability. These results suggest that, to improve PVC’s circular-economy potential, targeted interventions are required notably better waste segregation, enhanced recycling routes, additive substitution, and circular-design strategies.

#### 5.2.3. Qualitative/Performance Insights from Ontology Modelling

Ontology modelling results indicate that PLA is the lowest-carbon and lowest-energy polymer, with biodegradability confirmed through SPARQL queries. However, it has the lowest recycling rate (5–8%) (Table 7) and the highest cost (£2.5/kg). Accordingly, PLA is classified as a Biodegradable Polymer and a Low-Carbon Polymer, but also flagged as a High-Cost Material.

PET, though non-biodegradable, demonstrates the highest recycling rate (≈50%) and a relatively low cost (£1.1/kg). It is classified as a Recyclable Polymer within a 1.8 kg CO_2_ threshold, but not as low-carbon due to its higher virgin CO_2_ emissions (2.1–2.8 kg/kg). This distinction reflects literature values [36], where recycled PET corresponds to the lower figure (2.1 kg CO_2_/kg) and virgin PET to the higher (2.8 kg CO_2_/kg). Despite this variation, PET’s strong recyclability aligns with its circular-economy potential. PVC remains cost-effective (£1.2/kg) but shows higher emissions (1.9 kg CO_2_/kg), elevated embodied energy (81 MJ/kg), and only moderate recyclability (35%). These factors place PVC at a disadvantage compared to PET and PLA, highlighting the need for improved recycling infrastructure, additive substitution, and design-for-recycling measures.

Overall, the ontology-derived insights contribute to a knowledge-driven decision framework that supports both industry and policy in tailoring polymer circular-economy strategies. By balancing cost, sustainability, and recycling performance, these insights help define pathways for reducing the sector’s carbon footprint.

### 5.3. Simulation Findings: Material Circularity and Trade-Offs

A comparative simulation analysis was conducted using a comprehensive materials database to evaluate sustainability and recyclability trade-offs. Key parameters included embodied energy, CO_2_ emissions, recyclability, and material longevity. These metrics informed a calculated circularity score, indicating each polymer’s suitability for circular-economy adoption.

PLA emerged as the most sustainable polymer, showing the lowest embodied energy and carbon footprint. Its biodegradability and reduced greenhouse gas emissions position it as a strong candidate for environmentally conscious applications. PET demonstrated superior recyclability, with rates ranging from 50–80% depending on dataset and industrial practices. Although its emissions and energy demand are higher than PLA, its high recycling rate significantly enhances its circularity profile. In contrast, PVC, while widely used for its durability and affordability, performed worst in terms of sustainability. It required the highest energy input and achieved only moderate recyclability (around 50%), resulting in the lowest circularity score and highlighting the urgent need for improved recycling technologies and waste management.

Together, these results highlight the trade-offs between cost, performance, and environmental impact. While PLA is the most sustainable, PET remains the most recyclable, and PVC continues to face systemic barriers. Table 8 illustrates these differences and provide actionable insights for selecting polymers that best align with circular-economy goals.

**Notes:**

- Recycling rate for PET is often cited as 80% in industry, though it varies; the data used in the simulation aligns with the 50–80% range.
- The circularity score is qualitative, based on combined environmental and recyclability metrics derived from simulation and ontology modelling.
- Cost values are based on contemporary market approximations and a simulation dataset.

### 5.4. Case Study Insights: Recycled vs. Virgin PVC and PET

The industrial assessment of a UK PVC plant highlights practical challenges and strategic opportunities for integrating recycled and virgin polymers within circular-economy systems. Waste-stream analysis shows that pre-consumer off-cuts account for ~38% of total waste, followed by defective profiles (25%) and line rejects (20%). These upstream losses especially the mixing of PVC off-cuts with composite components such as rubber seals and adhesives reduce the purity and value of recyclates and constrain industrial reprocessing [46].

Quantitative and qualitative evidence demonstrate clear differences in recyclability between PET and PVC. PET benefits from established recycling infrastructure and higher recovery rates, whereas PVC recycling is impeded by complex additive packages and inadequate separation procedures. This disparity indicates the need for polymer-specific lifecycle and process interventions to improve material recovery and lower environmental impact.

Digital maturity at the facility is low, with limited use of simulation tools and real-time monitoring, consistent with the qualitative insights in Section 4.4. Nevertheless, staff receptivity to digitalisation is strong, with over 70% supporting adoption of simulation dashboards and digital-twin technologies.

Ontology-based modelling proved valuable in this context by integrating heterogeneous inputs (material-flow measurements, simulation outputs, and stakeholder insights) to support end-to-end decision-making. By linking operational data with sustainability criteria, the ontology enables more informed assessments of recyclate feasibility and guides practical measures improved material separation, design-for-disassembly, additive substitution, and digital process control that together can increase the circularity of both PVC and PET.

### 5.5. Mapping Research Gaps in PVC Sustainability

A bibliometric and thematic review was performed to identify prevailing research gaps in polymer sustainability within a circular-economy context. Visualisation of publication trends, keyword co-occurrence, and thematic clusters highlighted four priority areas:

1. Bio-based plasticizers
  The development and industrial integration of bio-based plasticizers for PVC remain underexplored. Key challenges include ensuring compatibility with PVC matrices, preserving performance through mechanical recycling, and assessing full life-cycle environmental impacts.
2. Chemical-recycling pathways
  Mechanical recycling dominates practice, but comprehensive models of chemical recycling for PVC are lacking. Research should evaluate energy requirements, emissions, scalability, and economic viability to determine where chemical routes can complement or replace mechanical processes.
3. AI-driven sorting and contamination detection
  Machine learning and computer-vision approaches for sorting and contamination detection show promise for improving recyclate quality. However, their practical integration with semantic decision frameworks and real-time process control remains nascent and requires further development and industrial validation.
4. Unified semantic frameworks for decision support
  There is no widely adopted, machine-readable semantic model that links polymer lifecycle data, additive chemistry, recycling routes, and sustainability metrics. This fragmentation impedes comprehensive, data-driven decision-making for polymer circularity.

These gaps define a strategic research agenda: develop integrated semantic frameworks, advance and model chemical-recycling technologies, accelerate AI-enabled sorting and process control, and harmonise metrics and regulatory standards. Addressing these priorities will support scalable, data-informed solutions that improve the sustainability and circularity of PVC and related polymers.

#### 5.5.1. Thematic and Bibliometric Analysis of Polymer Circular-Economy Research

##### Thematic Analysis

A systematic thematic review of recent literature and studies was performed to identify key themes in polymer CE research. Eight primary research themes were identified, as shown in Table 9.

The thematic analysis of key journal sources highlights the main research areas advancing the polymer circular economy: (i) development and standardisation of lifecycle sustainability metrics to guide material selection and environmental assessment; (ii) innovations in mechanical, chemical, and biological recycling to address contamination and complex waste streams; (iii) the emergence of ontology-based semantic frameworks that enable integrated decision-making through digital twins and linked-data systems; (iv) simulation-based trade-off analyses that balance performance against sustainability; (v) growing interest in bio-based and biodegradable polymers; and (vi) the strong influence of evolving regulations and standards on traceability and compliance. Industrial case studies and early AI-driven sorting applications further reveal persistent challenges, including data fragmentation and organisational resistance, which demand coordinated, multidisciplinary solutions.

##### Bibliometric Analysis with VOSviewer

A bibliometric analysis using VOSviewer version 1.6.20 was conducted to map keyword co-occurrence patterns and thematic clusters in polymer circular-economy research. Frequently keywords, their clustering, and bibliometric linkage strengths are presented in Table 10:

##### VOSviewer Network Diagram

**Figure 10:**
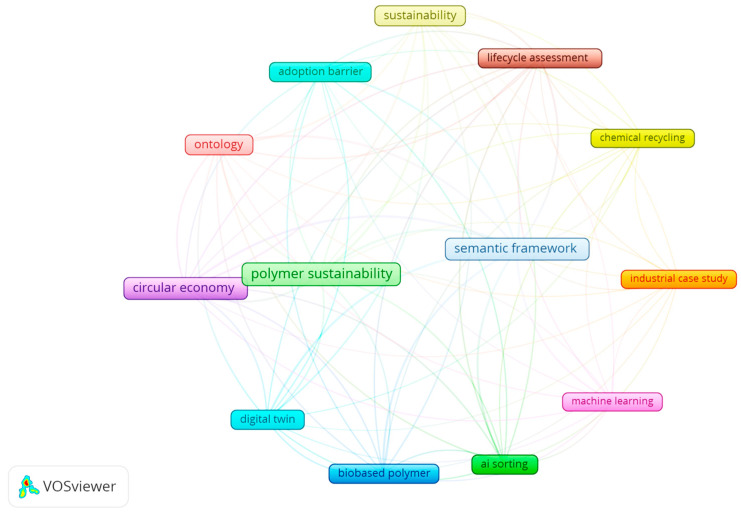
Bibliometric network mapping showing citation keywords and emerging research.

The resulting network diagram (Figure 10) shows the following:

- Core hotspots: polymer, sustainability, and circularity appear as the most significant and interconnected nodes.
- Digital/semantic linkages: terms such as digital twin and ontology/semantics are strongly associated with lifecycle concepts, reflecting the growth of digitalization and semantic integration.
- Network metrics: node size represents keyword frequency, while edge thickness reflects the strength of co-occurrence.

Two main findings emerge:

- Expanding digital and semantic cluster: ontology, digital twins, and AI applications are forming an emergent cluster that is beginning to close data gaps across lifecycle stages.
- Simulation as a central but under-deployed capability: simulation-driven decision-making and advanced modelling are recognised as crucial to implementation, though real-world deployment remains limited.

These thematic clusters emphasise the growing role of digital and semantic paradigms in achieving polymer circularity. Accordingly, the paper revisits the developed ontology model in the following section, exploring its integration with enterprise systems and digital twins to support real-time, scalable decision-making in circular-economy contexts.

Examination of the network mapping shows two primary outcomes. Firstly, that digital and semantic frameworks are an expanding cluster, with ontology, digital twin, and AI applications beginning to bridge data gaps. Secondly, simulation-driven decision-making and advanced modelling are recognised as central to practical implementation, although real-world deployment is limited. The identified theme clusters emphasise the increasing importance of digital and semantic paradigms in achieving polymer circularity goals. Based on these findings, this paper revisits the developed ontology model in Section 6 and explores how it can be integrated with enterprise systems and digital twins to support real-time, scalable decision-making in the circular economy.

## 6. Implementation Perspectives

The ontology, simulation screening, and industrial case study together indicate that a transition to a circular, sustainable polymer economy requires tight integration with existing digital infrastructure and a realistic appraisal of adoption challenges. This section considers the industrial viability of ontology-driven CE architectures, focusing on two pillars: (1) integration with ERP, IIoT, and digital twins, and (2) the challenges and opportunities of scaling these solutions.

### 6.1. Integration with ERP/IoT/Digital Twin Systems

#### Requirements for Semantic Integration

The ontology (OWL 2) enhanced with SWRL rules and SPARQL queries is con-ceived as a dynamic knowledge layer, not a static repository. For operational impact it should be embedded within the firm’s digital environment along three principal axes:

- ERP platforms: Map ontology classes (e.g., Material, WasteStream, Lifecycle Stage) to ERP master and transaction records so that procurement, inventory, production, and quality data are semantically enriched for downstream reasoning.
- IIoT and process-data integration: IIoT sensors (composition checks, process parameters, waste counters) generate high-resolution streams that can be mapped to ontology instances (observations/data properties) via semantic middleware or event brokers, enabling automated updates and provenance tracking.
- Digital-twin synchronisation: Synchronise live event streams and simulation outputs with digital twin models and the ontology to create a unified, real-time virtual representation of assets, lines, and products that supports simulation-driven decision-making.

### 6.2. Industry Adoption Challenges and Opportunities

Challenges

- Legacy data and silos: Much operational data remains in spreadsheets, bespoke databases, or paper logs. Converting these into ontology-conformant records requires substantial data-cleaning, harmonisation, and semantic annotation.
- Real-time performance and scalability: Reasoning engines must process large, continuous data flows and deliver timely inferences; production-scale deployment demands high-performance rule engines and semantic stores.
- Semantic granularity and data completeness: Decision reliability depends on inclusion of key attributes (additive content, batch lineage, waste composition); missing or coarse-grained records weaken inference quality.

Opportunities

- Closed-loop manufacturing: Real-time semantic inference can enable immediate decisions in waste streams (segregation, redirection, reuse), improving yield and circularity.
- Sector collaboration and standards: Adopting open semantic standards facilitates benchmarking, secure data sharing, and collaborative innovation across supply chains.

Practical rollout pathway

A pragmatic, staged approach reduces risk: (1) pilot ontology–ERP integration to align master data; (2) map IIoT sensors to ontology instances and validate data flows; (3) deploy digital twins synchronised with ontology and simulations; (4) scale toward cross-firm semantic interoperability. Successful implementation requires concurrent investments in change management, staff training, and cross-organisational governance.

## 7. Conclusions

### 7.1. Summary of Contributions

This study introduces an ontology-driven framework for modelling, analysis, and decision support of sustainable polymer systems, with a focus on polyvinyl chloride (PVC) and circular-economy (CE) applications. The principal contributions are:

- Ontology construction and semantic reasoning: A domain-specific ontology was developed and populated in Protégé (OWL 2), defining classes, object/data properties, and relationships needed to characterise polymer materials, recycling streams, processing steps, sustainability metrics, and environmental-impact parameters.
- Quantitative and qualitative trade-off analysis: ANSYS Granta EduPack datasets were used to simulate and rank polymers (PVC, PLA, PET) on mechanical, thermal, economic, and environmental criteria, supporting data-driven material selection and sustainability assessment.
- Knowledge graphs and inferred CE pathways: The ontology generates knowledge graphs that represent circularity flows, reveal waste-stream barriers, and expose cross-material relationships to support decision-making.

### 7.2. Limitations

Several limitations should be noted. The ontology, although built from industry and academic sources, can be extended to cover a broader range of polymer chemistries, emerging recycling technologies, and diverse regulatory contexts. However, industrial validation is based on a single UK PVC manufacturer; therefore, findings related to supply chains, waste profiles, and digital maturity may not apply to all sectors or regions.

### 7.3. Future Research Directions

Building on the ontology-based framework developed in this study, future research should prioritize the development of web-based e-manufacturing platforms that enable engineers to access, share, and simulate design and manufacturing data in real time [57]. Such platforms support interactive 3D simulation and demonstrations, thereby improving manufacturers’ decision-making. Building on these capabilities, subsequent work must address current digital and integration constraints and broaden the ontology to incorporate emerging polymer types (bio-based, degradable, and high-performance composites) and advanced recycling methods (for example, enzymatic and dissolution processes). Standardised sustainability indicators including toxicity, microplastic release, and social-impact metrics should also be embedded within the framework. A later phase should focus on developing and prototyping semantic digital twins that provide dynamic, two-way feedback between plant operations, lifecycle-analysis tools, and ontology-based decision agents. Integrating advanced AI will enable predictive analytics, anomaly detection, and closed-loop optimisation of recycling and manufacturing processes by leveraging historical knowledge-graph data. Together, these developments from web-based e-manufacturing to semantic modelling and digital twins will help bridge semantic polymer design and industrial-scale circular-economy implementation, enabling real-time, data-driven management of polymer lifecycles.

### 7.4. Concluding Statement

In conclusion, this research demonstrates that ontology-based modelling and semantic reasoning are effective tools for integrating data, increasing transparency, and producing actionable insights to advance circular-economy initiatives. Focusing on PVC as a case study, the study provides an ontology-based decision-support framework for sustainable polymer recycling, validated through a case study of a UK company and simulation analysis. By addressing technical and semantic gaps, the approaches developed are applicable to other engineered polymers and provide actionable guidance for material selection and future digitalisation of circular polymer systems, thereby supporting wider efforts in sustainable materials science and manufacturing.

## Figures and Tables

**Table 1:** Key simulation results from Ansys Granta EduPack Software [36].

Polymer	Key Strengths	Thermal & Mechanical Properties	Environmental Performance	Circularity & Cost Insights
PVC	High stiffness (3.3 GPa), 50 MPa strength	Tg = 80 °C, Thermal conductivity = 0.2 W/m·K	Virgin CO_2_: 2.1 kg, Recycled CO_2_: 1.2 kg	Cost-effective (£1.2/kg), moderate recycling rate (35%)
PET	Strongest (65 MPa), 2.9 GPa stiffness	Tg = 75 °C, higher thermal conductivity (0.24 W/m·K)	Highest Virgin Polymers CO_2_ (2.8 kg), lowest Recycled CO_2_ (0.9 kg)	High recyclability (50%), cost £1.1/kg
PP	Moderate strength, low stiffness (1.6 GPa)	Lowest Tg (−10 °C), moderate thermal conductivity (0.22 W/m·K)	Virgin CO_2_: 2.2 kg, Recycled: 1.4 kg	Low cost (£1/kg), moderate circularity (30%)
PLA	Comparable to PET (60 MPa), good strength	Tg = 60 °C, lowest thermal conductivity (0.13 W/m·K)	Lowest Virgin CO_2_ (1.5 kg), Recycled CO_2_: 0.8 kg	Compostable, high cost (£2.5/kg), only 5% recycling
PHA	Biodegradable, lower mechanical strength (1.5 GPa, 40 MPa)	Tg = 5 °C, low thermal conductivity (0.15 W/m·K)	High Virgin CO_2_ (2.65 kg), Recycled CO_2_: 1.5 kg	High cost (£3/kg), excellent circularity potential
PS	Stiff (3.2 GPa), moderate tensile strength (45 MPa)	Tg = 100 °C, thermal conductivity = 0.14 W/m·K	Highest energy use (90 MJ/kg), Recycled CO_2_: 2 kg	Non-biodegradable, low recycling rate (10%), low circularity

**Table 2:** Summary comparison of polymer properties [36].

Polymer	Strength & Modulus	Environmental Footprint	Recycling Rate (%)	Biodegradability	Cost (GBP/kg)	Circularity Rating
PET	High (65 MPa, 2.9 GPa)	Moderate CO_2_ (2.8/0.9)	50%	Non-biodegradable	£1.10	High
PVC	Strong (50 MPa, 3.3 GPa)	CO_2_ (2.1/1.2), Energy-efficient	35%	Non-biodegradable	£1.20	Moderate
PP	Medium (35 MPa, 1.6 GPa)	CO_2_ (2.2/1.4), low Tg	30%	Non-biodegradable	£1.00	Moderate
PLA	High (60 MPa, 3.0 GPa)	Low CO_2_ (1.5/0.8), low energy	5%	Compostable	£2.50	Emerging
PHA	Medium (40 MPa, 1.5 GPa)	Biogenic CO_2_ (2.65/1.5)	5%	Biodegradable	£3.00	High (Biobased)
PS	Moderate (45 MPa, 3.2 GPa)	Highest CO_2_ (2.9/2.0)	10%	Non-biodegradable	£1.10	Low

**Table 3:** Individual Polymers Inferences asserted from SWRL analysis [51].

Individual	Type	CO_2_ (kg CO_2_e/kg)	Embodied Energy (MJ/kg)	Recyclability	Biodegradable	Cost (GBP/kg)	Thermal Resistance (°C)	Mechanical Strength (MPa)	Sustainability Verdict
PLA_01	PolymerGrade	1.2	55.0	Medium	true	1.5	60.0	65.0	“Very Good”
PET_01	PolymerGrade	1.6	70.0	High	false	1.0	70.0	60.0	“Good”
PVC_01	PolymerGrade	2.0	80.0	Medium	false	1.2	60.0	50.0	“Moderate”

**Table 4:** SWRL rules for classification of polymers [51].

Rule Name	Rule (SWRL)
BiodegradablePolymer	Polymer(? p) ^ isBiodegradable(? p, true) -> BiodegradablePolymer(? p)
RecyclablePolymer	Polymer(? p) ^ hasRecyclability(? p, “High”) -> RecyclablePolymer(? p)
RecommendedMaterial	Polymer(? p) ^ hasSustainabilityVerdict(? p, “Very Good”) -> RecommendedMaterial(? p)
LowCarbonPolymer	Polymer(? p) ^ hasCO2Emissions(? p, ? e) ^ swrlb:lessThan(? e, 1.8) -> LowCarbonPolymer(? p)

Note: In these SWRL rules, (? p) ^ refers to a polymer instance and (? p,? e) refers to a data property such as CO_2_ emissions. These rules are implemented in Protégé using Semantic Web Rule Language (SWRL) logic to enable material classification within the ontology. The variables are applied to infer new classifications.

**Table 5:** SPARQL Queries comparative analysis of polymers [51].

Polymer	CO_2_ (kg CO_2_e/kg)	Embodied Energy (MJ/kg)	Recyclability	Biodegradable	Sustainability Verdict	Inferred Classes (From SWRL Rules)
PVC_01	2.0	80.0	Medium	No	Moderate	
PLA_01	1.2	55.0	Medium	Yes	Very Good	Biodegradable Polymer, Recommended Material, LowCarbonPolymer
PET_01	1.6	70.0	High	No	Good	Recyclable Polymer, Low-Carbon Polymer

**Table 6:** Consolidated comparative analysis of these SPARQL assertions for PET, PLA, and PVC [51].

Data Property	PET_01	PLA_01	PVC_01
has Recyclability	High	Medium	Medium
has Sustainability Verdict	Good	Very Good	Moderate
is Biodegradable	False	True	False
has Mechanical Strength	60.0	55.0	60.0
has Thermal Resistance	70.0	60.0	80.0
has CO2Emissions	1.6	1.2	2.0
has Embodied Energy	70.0	55.0	80.0
has Cost	1.0	1.5	1.2

**Table 7:** SPARQL Cost and related metrics comparative analysis [51].

Polymer	Cost (GBP/kg)	Recycling Rate (%)	CO_2_ (kg CO_2_e/kg)	Embodied Energy (MJ/kg)
PVC	£1.20	35%	1.9	81
PET	£1.10	50%	2.1	70
PLA	£2.50	8%	0.6	26

**Table 8:** Simulation findings: material circularity and trade-off metrics for PVC, PLA, and PET.

Polymer	Embodied Energy (MJ/kg)	CO_2_ Emissions (kg CO_2_e/kg)	Recycling Rate (%)	Bio- Degradability	Cost (GBP/kg)	Circularity Score	Key Insights	References
PLA	55	1.2	5–8	Yes	2.5	Highest	Lowest energy use and CO_2_ emissions, biodegradable but low recycling rate, and high cost	[3]
PET	70	1.6—2.8	50–80	No	1.1	High	High recyclability, moderate embodied energy, not biodegradable, cost-effective for closed-loop recycling	[23]
PVC	80	1.9—2.1	35–50	No	1.2	Moderate	Widely used industrially, highest energy demand, medium recyclability, non-biodegradable; recycling improvements needed	[23]

**Table 9:** Thematic analysis of key themes extracted from the literature.

Theme	Description	References
Lifecycle Sustainability Metrics	Development and harmonisation of metrics such as carbon footprint, energy use, recyclability, biodegradability, and eco-labelling to assess polymer environmental impact and circularity.	[3,52,53]
Advanced Recycling and Valorisation	Innovations in mechanical, chemical, and biological recycling approaches addressing contamination, mixed waste streams, upcycling, and waste valorisation challenges in polymers such as PVC and PET.	[2,20,54]
Ontology-Based Semantic Frameworks	Use of OWL 2 ontologies, SWRL rule engines, and SPARQL querying to create integrated models enabling semantic interoperability, real-time decision support, and CE data integration via ERP, IoT, and digital twins.	[23,24,38]
Simulation-Driven Trade-Off Analysis	Application of lifecycle assessment tools (e.g., ANSYS Granta EduPack) and scenario simulations to optimise polymer selection, balancing mechanical properties, environmental impact, and cost.	[23]
Bio-Based and Biodegradable Polymers	Research on biobased plastics (PLA, PHA) and biodegradable alternatives aimed at reducing fossil resource dependence and improving end-of-life sustainability.	[3,53,55]
Regulatory and standardisation Challenges	Exploration of evolving sustainability regulations, Extended Producer Responsibility (EPR), digital product passports, and the need for harmonised standards for sustainable polymer data reporting.	[24,54,56]
Artificial Intelligence & Data-Driven Sorting	Emerging AI-enabled technologies for contamination detection, material identification, and automated waste sorting, when integrated with semantic tools, remain at early development stages.	[24,48]
Industrial Case Studies and Adoption Barriers	Empirical validation of semantic and circular-economy frameworks using industrial data; barriers include data fragmentation, organisational resistance, and knowledge gaps in applying CE at scale.	[2,23]

**Table 10:** Co-occurrence keyword data.

Keyword	Occurrences	Links	Cluster
Polymer sustainability	120	540	1
Circular economy	95	500	1
Sustainability	85	470	1
Ontology	70	410	2
Digital twin	65	390	2
Lifecycle assessment	55	320	1
Machine learning	45	280	2
Chemical recycling	40	260	1
Recycling technology	91	480	1
AI sorting	59	290	1
Bio-based polymers	61	340	1
Industrial case study	27	170	1
Semantic framework	30	210	1
Adoption barriers	58	250	1

## Data Availability

The original contributions presented in this study are included in the article. Further inquiries can be directed to the corresponding author(s).
